# The Experiences of Homeless Youth When Using Strengths Profiling to Identify Their Character Strengths

**DOI:** 10.3389/fpsyg.2019.02036

**Published:** 2019-09-24

**Authors:** Sam J. Cooley, Mary L. Quinton, Mark J. G. Holland, Benjamin J. Parry, Jennifer Cumming

**Affiliations:** ^1^School of Sport, Exercise and Rehabilitation Sciences, University of Birmingham, Birmingham, United Kingdom; ^2^Department of Sport and Health, Newman University, Birmingham, United Kingdom

**Keywords:** strength-based approach, idiographic and nomothetic approaches, mixed-method data, psychological well-being, hard-to-reach populations

## Abstract

Individuals, particularly those considered “hard-to-reach,” often engage well with assessment tools that involve active dialogue and the co-construction of knowledge. Strengths profiling is one such tool that enables a person-centered and autonomy supportive approach to the identification of character strengths. Strength profiling is an adaptation of performance profiling used in sport psychology, which has not yet been utilized in broader psychological research or clinical practice. Supporting an individual by raising awareness of their personal character strengths is an effective and growing mechanism for promoting psychological well-being. Strengths profiling involves several stages of exploring, defining, and assessing character strengths, leading to the identification of signature strengths and goals for future development. Informed by personal construct theory, the present study explored the experiences of homeless young people living in sheltered accommodation (*N* = 116), when using strengths profiling at the start and end of a 10-week, strengths-based intervention. Mixed-method data was obtained from the strengths profiles, questionnaires measuring resilience, self-worth, and well-being, and diary entries. Findings revealed a rich array of character strength terminology and individual meanings. Participants found strengths profiling to be highly engaging, particularly due to their active role in strength identification, which prompted interesting and meaningful reflections on character strengths that were pertinent to them. Participants felt their signature strengths were vital protective factors within their lives and strengths profiles were correlated with resilience, self-worth, and well-being. Character strengths and resilience were also significantly and meaningfully improved pre/post-intervention, providing support for the use of strengths profiling as a tool for monitoring change in character strength perceptions. Overall, this study demonstrates the utility and versatility of strengths profiling as a new method in the discipline of positive psychology and strengths-based research and applied practice.

## Introduction

A strengths-based approach involves identifying areas of life in which a person succeeds, and the positive characteristics they demonstrate in doing so (Park and Peterson, 2009). This form of positive psychology has the potential to benefit everyone, as time spent focusing on our strengths is known to foster hope and psychological growth (Smith, 2006). Becoming more aware of our personal character strengths (e.g., creativity, perseverance, kindness, and self-regulation) can enable us to increase our use of these strengths (Schutte and Malouff, 2019), build social support networks (Gillham et al., 2011), navigate major life transitions (Shoshani and Slone, 2013), succeed in work and education (Morales, 2010), experience greater life satisfaction (Schutte and Malouff, 2019), and buffer against mental illness (Seligman et al., 1999; Huta and Hawley, 2010).

Strengths-based interventions may be of particular importance in homeless young people, who generally report having fewer character strengths and instead perceive themselves as “a list of problems” in need of “fixing” (Bender et al., 2007; Heinze, 2013). In feeling stigmatized by society, many of these young people disengage when support is offered, especially when that support is focused on their deficits (Smith, 2006; Ungar, 2006). Studies of young people living in sheltered accommodation find those who report having greater internal assets (e.g., positive values and life perspectives, individual strengths, and social competencies) are more likely to report lower levels of distress, more positive health behavior, and greater life satisfaction and resilience (Heinze, 2013; Thompson et al., 2016). Awareness of these character strengths also support homeless young people in their re-engagement with society, such as through gaining employment, education, and other means of independence (Lindsey et al., 2000). Informed by previous research, interventions that support high-risk youth in recognizing and developing their character strengths are now recommended for achieving significant life turning points (Thompson et al., 2016).

Most character strength interventions begin by administering a tool that helps individuals to identify their “signature” character strengths, before these signature strengths are applied and further developed in a variety of behavioral experiments (Quinlan et al., 2012). Common tools for identifying character strengths in young people include the Clifton Youth Strengths Explorer Assessment (CYSE; The Gallup Organization, 2006), Realise2 (Linley and Stoker, 2012), and the Developmental Assets Profile (DAP; Search Institute, 2005). Arguably one of the most frequently used and validated tools is the Values in Action Inventory of Strengths (VIA-IS; Peterson et al., 2005; Park et al., 2006), which has also been applied to homeless populations (Tweed et al., 2012). The VIA-IS is based on a framework of 24 groups of character strengths recognized across world cultures (Table 1; Peterson and Seligman, 2004). Character strengths are defined by the VIA as the positive parts of our personality that impact how we think, feel and behave, which can benefit both the individual and their society when used effectively (Peterson and Seligman, 2004). The 24 areas of character strength within the VIA make up six broader categories referred to as core virtues (wisdom and knowledge, courage, humanity, justice, temperance, and transcendence), which are supported through conceptual testing (Ruch and Proyer, 2015). The 24 character strength groups can be assessed through various adapted survey instruments and structured interviews; however, they are most commonly measured using the standard 240-item VIA-IS survey (Peterson et al., 2005). After assessing these 24 areas of character strength, the VIA-IS reveals an individual’s strongest characteristics (i.e., signature strengths). The VIA-IS is widely supported across a range of cultural groups, making the VIA one of the most established frameworks in positive psychology (Park et al., 2006; McGrath, 2015).

**TABLE 1:** The VIA classification of strengths.

**Core virtue**	**Character strengths**	**Example**
Wisdom and knowledge (cognitive strengths that entail the acquisition and use of knowledge)	Creativity	[Originality, ingenuity]: Thinking of novel and productive ways to conceptualize and do things; includes artistic achievement but is not limited to it
	Curiosity	[interest, novelty-seeking, openness to experience]: Taking an interest in ongoing experience for its own sake; finding subjects and topics fascinating; exploring and discovering
	Judgment	[Critical thinking]: Thinking things through and examining them from all sides; not jumping to conclusions; being able to change one’s mind in light of evidence; weighing all evidence fairly
	Love of learning	Mastering new skills, topics, and bodies of knowledge, whether on one’s own or formally; obviously related to the strength of curiosity but goes beyond it to describe the tendency to add systematically to what one knows
	Perspective	[Wisdom]: Being able to provide wise counsel to others; having ways of looking at the world that make sense to oneself and to other people
Courage (emotional strengths that involve the exercise of will to accomplish goals in the face of opposition, external or internal)	Bravery	Not shrinking from threat, challenge, difficulty, or pain; speaking up for what is right even if there is opposition; acting on convictions even if unpopular; includes physical bravery but is not limited to it
	Perseverance	[Persistence, industriousness]: Finishing what one starts; persisting in a course of action in spite of obstacles; “getting it out the door;” taking pleasure in completing tasks
	Honesty	[Authenticity, integrity]: Speaking the truth but more broadly presenting oneself in a genuine way and acting in a sincere way; being without pretense; taking responsibility for one’s feelings and actions
	Zest	[Vitality, enthusiasm, vigor, energy]: Approaching life with excitement and energy; not doing things halfway or halfheartedly; living life as an adventure; feeling alive and activated
Humanity (interpersonal strengths that involve tending and befriending others)	Love	Valuing close relations with others, in particular those in which sharing and caring are reciprocated; being close to people
	Kindness	[Generosity, nurturance, care, compassion, altruistic love, “niceness”]: Doing favors and good deeds for others; helping them; taking care of them
	Social intelligence	[Emotional intelligence, personal intelligence]: Being aware of the motives and feelings of other people and oneself; knowing what to do to fit into different social situations; knowing what makes other people tick
Justice (civic strengths that underlie healthy community life)	Teamwork	[Citizenship, social responsibility, loyalty]: Working well as a member of a group or team; being loyal to the group; doing one’s share
	Fairness	Treating all people the same according to notions of fairness and justice; not letting personal feelings bias decisions about others; giving everyone a fair chance
	Leadership	Encouraging a group of which one is a member to get things done and at the time maintain time good relations within the group; organizing group activities and seeing that they happen
Temperance (strengths that protect against excess)	Forgiveness	Forgiving those who have done wrong; accepting the shortcomings of others; giving people a second chance; not being vengeful
	Humility	Letting one’s accomplishments speak for themselves; not regarding oneself as more special than one is
	Prudence	Being careful about one’s choices; not taking undue risks; not saying or doing things that might later be regretted
	Self-regulation	[Self-control]: Regulating what one feels and does; being disciplined; controlling one’s appetites and emotions
Transcendence (strengths that forge connections to the larger universe and provide meaning)	Appreciation of beauty and excellence	[Awe, wonder, elevation]: Noticing and appreciating beauty, excellence, and/or skilled performance in various domains of life, from nature to art to mathematics to science to everyday experience
	Gratitude	Being aware of and thankful for the good things that happen; taking time to express thanks
	Hope	[Optimism, future-mindedness, future orientation]: Expecting the best in the future and working to achieve it; believing that a good future is something that can be brought about
	Humor	[Playfulness]: Liking to laugh and tease; bringing smiles to other people; seeing the light side; making (not necessarily telling) jokes
	Spirituality	[Faith, purpose]: Having coherent beliefs about the higher purpose and meaning of the universe; knowing where one fits within the larger scheme; having beliefs about the meaning of life that shape conduct and provide comfort

*The above definitions are from the VIA website: https://www.viacharacter.org/character-strengths.*

These standardized frameworks and instruments have many benefits as they enable researchers and practitioners to categorize an individual’s strengths into a universal framework. This categorization helps to make sense of a person’s positive attributes in a common language, administer tools to large groups, and easily compare between groups (Peterson and Seligman, 2004; Smith, 2006). In this regard, these instruments can be classified as nomothetic, as they are concerned with universal theories and identifying aggregates and commonalities within and between populations (Runyan, 1983; Grice, 2004).

Whilst nomothetic instruments such as the VIA-IS contribute greatly to the positive psychology movement, there are certain situations where administering a nomothetic survey is problematic. These situations include “hard-to-reach” populations, such as homeless young people, who are typically more effectively engaged through active dialogue and co-construction of knowledge, rather than a more prescriptive and often solo “pen and paper” survey (e.g., Powers and Tiffany, 2006; Gomez and Ryan, 2016). Even when these survey instruments are accepted, there is an inherent risk of inaccuracy resulting from the individual nuances in the way survey items are interpreted and responded to Grice (2004). As described in Kelly’s (1955) Personal Construct Theory (PCT), every individual has their own unique lens, or personal set of constructs, through which they view life. These personal constructs are influenced by a number of corollaries, such as our life experiences that shape how we perceive and interpret new events (experience corollary). Whilst some of our personal constructs will be developed and shared with others (commonality corollary), many will be specific to the individual (individuality corollary). As a result of our unique constructs, any two individuals are likely to have, (a) unique sets of character strengths they consider important, (b) different labels to represent the same strength, and (c) different meanings attached to similar labels. These personal constructs are evident in disadvantaged youth who, for example, are found to use unique language (or “street slang”) when describing their character strengths, as well as having individual pathways to resiliency and well-being (Bender et al., 2007; Nott and Vuchinich, 2016). As the VIA-IS, and similar nomothetic instruments, were designed to capture the most commonly recognized terms across world cultures, they are considered “neither exclusive nor exhaustive” (p. 13) and do not capture the unique language and associated meanings within the more marginalized subgroups of society (Peterson and Seligman, 2004).

An alternative, or supplement, to the more standardized, nomothetic survey instruments is the ideographic approach, which seeks to understand the unique way an individual interprets reality (Runyan, 1983; Grice, 2004). Ideographic approaches such as qualitative interviews, repertory grids (Kelly, 1955), and the Q-sort method (Stephenson, 1953) are more appropriate for capturing personal constructs than nomothetic alternatives. Indeed, studies have generally found weak correlations when comparing findings from ideographic and nomothetic measures, demonstrating that the two approaches can sometimes paint very different pictures of an individual (e.g., Schiller et al., 1995; Robazza et al., 2000; Van Kampen, 2000; Grice, 2004).

Aside from qualitative interviews, ideographic tools such as repertory grids and Q-sort rarely feature in published research in youth development and applied practice (for examples of their application see Shepard and Marshall, 1999; Borell et al., 2003; Shek, 2012). One exception, however, is an ideographic tool called performance profiling (Butler and Hardy, 1992), which is popular in youth development within the sporting context. This tool was introduced in the early 1990s to help athletes become more aware of their strengths and areas for development in relation to their sporting performance (Butler and Hardy, 1992; Jones, 1993). Performance profiling stemmed from Kelly’s PCT and repertory grids, and a number of studies have demonstrated its validity and reliability in sport, including predictive validity (Doyle and Parfitt, 1996), construct validity (Doyle and Parfitt, 1997), its positive impact on intrinsic motivation (Weston et al., 2011), and its sensitivity to change pre/post-intervention (for reviews see Gucciardi and Gordon, 2009; Weston et al., 2013). The benefits of performance profiling, however, have yet to be realized outside the physical activity context, where the approach could easily be adapted to identify character strengths related to life in general rather than those specific to sport and exercise performance.

Performance profiling is used with individuals or groups and involves the athlete self-selecting a list of characteristics that are meaningful to their engagement in their sport, based on their experience and in their language, usually with the guidance of a coach and/or sport psychologist. After providing a definition for each characteristic, the athlete gives each one a score out of 10 for how important it is to them or to a particular goal they wish to achieve (1 = *not so important*, 10 = *extremely important*), their ideal level, and their current level (1 = *poor*, 10 = *exceptional*). A discrepancy score is then calculated for each characteristic by subtracting the current level from the ideal, and multiplying by importance (Jones, 1993). A larger discrepancy score represents a characteristic that is important to the athlete but reflects a larger gap between their current ability and where they would like to be, whereas a lower discrepancy score represents greater satisfaction. In contrast to a typical nomothetic survey that measures current ability in a predetermined set of characteristics, the performance profile involves a more qualitative enquiry into meaning, importance, current ability, and future goals, on an unlimited range of characteristics, which can then be assessed using quantitative or qualitative methods (Weston et al., 2013).

Through the co-creation of knowledge, performance profiling may also help to address the power imbalance sometimes observed between a young person and a researcher or practitioner (Skelton, 2008; Harris et al., 2014). During performance profiling, the young person is given autonomy over identifying and defining their own list of characteristics on which to score themselves (Butler and Hardy, 1992). In playing a more active and autonomous role in the process of strength identification, the young person is also likely to feel more empowered, self-regulated, and intrinsically motivated (i.e., the very goal of most youth development programs; Deci and Ryan, 1985; Zimmerman, 2002; Weston et al., 2011). Indeed, previous research suggests that successful character strength interventions are those that satisfy one’s basic psychological needs and thus foster the self-determination to actually use the strengths identified (Quinlan et al., 2012). In adopting a person-centered and autonomy supportive approach to identifying strengths, performance profiling blends multiple psychological frameworks and movements, including strengths-based and positive psychology, personal construct psychology, and self-determination theory (Kelly, 1955; Deci and Ryan, 1985; Seligman, 1991; Seligman and Csikszentmihalyi, 2000). This approach may therefore be particularly welcomed by disadvantaged young people, who regularly find themselves in disempowering situations where they lack control (Gomez and Ryan, 2016).

The aim of the present study was to explore the experiences of homeless young people when taking part in performance profiling. The name performance profiling was changed to strengths profiling to reflect its adaption to the identification of character strengths related to life in general as opposed to its originally intended context of sporting performance (Butler and Hardy, 1992). Strengths profiling was used as part of a broader youth development program called My Strengths Training for Life^TM^, which comprises a series of experiential activities that aim to develop life skills in areas such as problem solving, self-regulation, and working with others, as a way of enhancing resilience, self-worth, and well-being in homeless young people, and ultimately supporting their re-engagement with society.

The present study explored several areas of strengths profiling in homeless young people: the aspects of strengths profiling that best supported engagement; the types of character strengths that are identified by homeless young people and how these characteristics compare to those in existing nomothetic frameworks (i.e., the VIA); whether discrepancy scores calculated through strengths profiling are related to brief nomothetic measures of resilience, well-being, and self-worth; and whether strengths profiling detects change following a youth development intervention. These questions were addressed using an integrated mixed-method approach.

## Materials and Methods

### Participants

Of the 118 young people who participated in the study, ages ranged from 16–24 years (*M* = 19.90 years; *SD* = 2.28), 56.9% were female, 41.4% male, and 1.7% transgender. The majority described their ethnicity as White (60.7%), followed by multiple ethnic groups (18.8%), Black (15.2%), and Asian (4.5%). The majority were born in the United Kingdom (87.6%) and spoke English as their first language (95.6%). Those born outside the United Kingdom had lived in the United Kingdom for an average of 10 years (*SD* = 6 months). Participants had either been homeless or at risk of homelessness, and as a result, had been living in supported accommodation for between a few days and 3.5 years intermittently (*M* = 7 months, *SD* = 6 months). In response to their employment status, 39.6% described themselves as unemployed, 22.5% in full time education, 9.9% in paid employment, 8.1% on an apprenticeship scheme, and 16.2% as being unable to work. A number of participants (17.3%) also disclosed having a learning disability.

### Study Context

Participants of the present study were living in supported accommodation provided by the United Kingdom Midlands-based youth homeless charity, St Basils^1^. Young people at St Basils were encouraged by their support workers to voluntarily take part in a 10-week, strengths-based, youth development program called My Strengths Training for Life^TM^ (MST4Life^TM^), especially if they were not engaged in education, employment, or training (NEET). MST4Life^TM^ is delivered in groups of between 6 and 12 young people and is facilitated by a multidisciplinary team, including academics (all authors; who specialize in sport and exercise psychology, outdoor education, youth development, and psychological skills training), support workers and peer mentors at St Basils, and a consultant Clinical Psychologist.

Phase 1 of MST4Life^TM^ involves 10 weekly sessions of community-based, experiential learning. During this time, the group meets weekly for between 2 and 4 h for activities designed to introduce and develop mental techniques, skills, and qualities (e.g., problem solving, action planning, managing emotions, working in a team, self-awareness, mobilizing social support, and building self-confidence). These activities, such as organizing a charity cake sale, are designed to provide opportunities for the self-discovery of strengths, which is prompted through guided reflections led by the mental skills facilitator (in accordance with Gibbs’ (1988) reflective cycle). At the end of the 10-weeks, Phase 2 involves a 4-day outdoor adventure residential in the Lake District, where participants can experiment and further develop what is learnt in a novel and challenging environment (for further details of MST4Life^TM^, see Cumming and Anderson, 2016). Participants of MST4Life^TM^ are offered a wide range of research tools (both ideographic and nomothetic) as a way of maximizing their ability to be heard within program evaluations.

### Measures

#### Strengths Profile

The strengths profiling exercise is a fundamental part of MST4Life^TM^ and was completed in seven stages during a single 60–90 min group session. Step 1 involved a group discussion about the personal qualities and characteristics that are important in life. Following a strengths-based approach, the facilitator typically asked the group to think about their own experiences and the positive characteristics that help them to thrive or cope with life’s challenges. During this process, the facilitator would probe for additional meaning and sometimes challenge assumptions to encourage deeper and broader reflections. At times, additional meaning would also be generated through reflecting on the polar opposites of characteristics (e.g., happiness-sadness), which together is known as a “construct” in personal construct theory (Kelly, 1955). Once a detailed list of desirable characteristics had been produced, each member of the group was provided with a strengths profile worksheet (see Appendix A).

In Step 2, individuals were invited to list, on their worksheet (Appendix A), the characteristics that were particularly meaningful to them. Participants were free to list as many or as few characteristics as they wanted (i.e., further sheets were offered if required). It was emphasized that they could use any terminology they wished and that their list could contain characteristics from the existing list produced in the group discussion or any of their own that may not have been mentioned. In reference to a particular construct (e.g., happiness-sadness), the young people were asked to use the positive pole within their list of characteristics. It was also suggested that the list of characteristics might contain a combination of existing strengths as well as desirable characteristics young people might like to work toward. To promote ownership, the young person was encouraged to share what each characteristic meant to them, using examples if possible (either in writing or via a discussion with the facilitators, who maintained reflective diaries).

In Steps 3 to 5, the young person was asked to give each characteristic a score out of 10 in three different areas: how important each characteristic was within their lives (“importance;” 1 = *not so important*; 10 = *extremely important*), what, realistically, would be a satisfactory level for them to reach in relation to this characteristic (“ideal level;” 1 = *poor*; 10 = *excellent*), and where they were currently (“current level;” 1 = *poor*; 10 = *excellent*). In step 6, a discrepancy score was calculated for each characteristic by subtracting the current level from the ideal level and multiplying by importance (Jones, 1993). For example, if the ideal level was 10/10, current level was 7/10, and importance was 9/10, the discrepancy score would be: (10 – 7) × 9 = 27.

In the final step, the young person entered their discrepancy scores into a bar graph and reflected on their profile individually with the facilitator, such as what their greatest strengths were and how these strengths could be applied in a way that would support their areas of higher discrepancy. In an extension to the original performance profiling technique, the strengths profiling process was also informed by Solution-focused (Brief) Therapy (SFBT; De Shazer and Dolan, 2012) and strengths-based cognitive-behavioral therapy (Padesky and Mooney, 2012). Discussions therefore centered around establishing personal pathways to resilience and identifying individualized goals for the upcoming program activities.

#### Resilience

Resilience was measured using the 10-item Connor-Davidson Resilience Scale (CD-RISC; Campbell-Sills and Stein, 2007). Following the stem, “Over the past month I have felt that…,” participants rate statements such as “I am able to adapt to change…,” between 1 (not at all true) and 5 (true nearly all of the time). An average is then calculated across the 10 items. Satisfactory validity evidence has been provided for this scale (Campbell-Sills and Stein, 2007). Cronbach’s alphas were 0.90 at baseline and 0.90 postcourse.

#### Self-Worth

The 13-item Self Description Questionnaire III (Marsh and O’Neill, 1984) was used to measure self-worth. Participants rated statements such as “Overall, I have pretty positive feelings about myself” on a scale from 1 (false) to 5 (true). A number of items are reverse coded before an average is calculated. Previous validity evidence supports the psychometric properties of this scale (Marsh and O’Neill, 1984). Cronbach’s alphas were 0.90 at baseline and 0.91 postcourse.

#### Well-Being

The EPOCH Measure of Adolescent Well-being (Kern et al., 2016) was used to measure five subscales of well-being: Engagement (e.g., “I get completely absorbed in what I’m doing”), Perseverance (e.g., “I finish whatever I begin”), Optimism (e.g., “I am optimistic about my future”), Connectedness (e.g., “When I have a problem, I have someone who will be there for me”), and Happiness (e.g., “I feel happy”). Each subscale comprises four items and an average is calculated for each. Acceptable validity evidence has been provided (Kern et al., 2016). Cronbach’s alphas at baseline were 0.81, 0.81, 0.85, 0.80, and 0.82, respectively, and 0.83, 0.84, 0.78, 0.85, and 0.86 at postcourse, respectively.

### Procedure

The participants in the present study (*N* = 118) were a subsample recruited from 307 young people who had engaged with MST4Life^TM^ over a 2-year period between April 2015 and April 2017. As MST4Life^TM^ for the most part was an open group, not all young people attended every session. This subsample of 118 therefore comprised only those who attended the baseline strengths profiling session at the start of their program and had opted to complete a short baseline questionnaire the week before (measuring resilience, self-worth, and well-being).

Within this subsample of 118 participants, 30% (*n* = 35) were invited, using purposive sampling, to complete a semi-structured diary room entry about their character strengths and how they felt about the strengths profiling activity. The video diary room approach introduced by Cooley et al. (2014) was adapted to give participants the option of recording their entries orally (video camera or Dictaphone) or in written form, as well as the option of being alone in the diary room, with a researcher, or with their peers.

In Week 10, at the end of Phase 1 of MST4Life^TM^, 39% of the participants (*n* = 46) attended the session and were provided with their list of characteristics from their initial strengths profile and invited to re-score themselves. During this session, participants also completed a short post-program questionnaire again measuring their resilience, self-worth, and well-being.

In summary, 118 young people attended the relevant baseline questionnaire and strengths profiling sessions to be included as participants. Of this sample, 30% completed a diary entry after strengths profiling, and 39% completed a post-intervention strengths profile and questionnaire. Facilitator reflective diaries were also collected from each of the 32 separate strengths profiling sessions that were facilitated during the course of the study. Facilitators (*n* = 4; SC, MQ, MH, and BP) ran the sessions either on their own or as a group, which resulted in a total of 78 journal entries.

### Data Analysis

#### Quantitative

Data was manually entered into SPSS (Version 22.0) before being cleaned, screened, and checked for outliers and normality (Tabachnick and Fidell, 2013). Missing items were found to randomly occur in <1% of the data and were therefore replaced using the MCAR test (Little, 1988; as recommended by Tabachnick and Fidell, 2013). Parametric and non-parametric tests were used comprising *t*-test, Chi-Square, bivariate correlation, and analysis of variance (ANOVA).

#### Qualitative

Diary room entries were on average 450 words in length (total = 15,750 words) and facilitator reflective notes were on average 230 words in length (total = 17,940 words). Data from both sources were analyzed in one single thematic analysis, following an iterative process of data familiarization, coding of raw data, and the inductive sorting of codes into themes (Braun and Clarke, 2013). Continuous note taking and referring back to raw data was used and the wider research team was consulted on several occasions to critically appraise tentative themes. The resulting themes were prevalent across both the participant diary room data and facilitator data.

To explore whether the character strengths identified through strengths profiling could be transformed into the VIA framework (Peterson and Seligman, 2004), each character strength identified by each participant was deductively categorized, if applicable, into the closest fitting VIA group of character strengths (i.e., the VIA framework was the codebook). This categorization was based on the participants’ individual meaning provided; for example, if a participant used the label “team-work,” but instead of describing it according to the VIA definition of team-work, described it as “doing favors and good deeds for others” (i.e., the VIA definition for “kindness”), the character strength was coded as “kindness”. Two researchers (SC and a research assistant) independently categorized 80% of the profiles, with 91.16% agreement. Coding was then presented to all named authors to review the categorization with a small number of characteristics (2.30%) re-categorized based on these discussions, suggesting a high degree of consensus. A final 20% of data was categorized by SC and a research assistant, after consensus was reached, which resulted in no further anomalies.

## Results

### Preliminary Analysis

Independent samples *t*-tests and Chi-Square tests revealed no significant or meaningful differences at baseline between those who attended only the baseline strengths profiling session (*n* = 69) and those who also attended the end-of-program session where strengths profiling was completed a second time (*n* = 49) (*p* > 0.05; age, gender, ethnicity, duration of homelessness, employment status, resilience, self-worth, and well-being).

Correlational analyses were used as an indicator of whether the component scores of strengths profiling (i.e., importance, ideal level, current level) were reflective of distinct constructs. This analysis did indeed indicate distinct constructs with correlations between importance, ideal, and current levels ranging from non-significant to moderately sized positive correlations (Table 2). Importance, ideal level, and current level were significantly correlated with the discrepancy score, with current level showing the strongest relationship. Baseline correlations were largely the same between the full sample and subsample who went on to complete a post-intervention measure. Correlations from baseline to post-intervention also remained similar, apart from the correlation between ideal and current level becoming significant and moderate in size, and the correlation between ideal level and discrepancy scoring becoming non-significant.

**TABLE 2:** Correlations between strength profile subsections.

	**1**	**2**	**3**	**4**
**(a) Baseline for the full sample (*n* = 118).**				
(1) Importance	1			
(2) Ideal level	0.29^∗∗^	1		
(3) Current level	0.14	0.18	1	
(4) Discrepancy score	0.24^∗∗^	0.39^∗∗^	–0.80^∗∗∗^	1
**(b) Baseline for the subsample who completed a post-intervention measure (*n* = 49).**				
(1) Importance	1			
(2) Ideal level	0.25	1		
(3) Current level	0.05	0.18	1	
(4) Discrepancy score	0.34^∗^	0.38^∗∗^	–0.79^∗∗∗^	1
**(c) Post-intervention subsample (*n* = 49).**				
(1) Importance	1			
(2) Ideal level	0.44^∗∗^	1		
(3) Current level	–0.04	0.40^∗∗^	1	
(4) Discrepancy score	0.31^∗^	0.14	–0.85^∗∗∗^	1

*^∗^*p* < 0.05; ^∗∗^*p* < 0.01; and ^∗∗∗^*p* < 0.001.*

### Main Analysis

#### Qualitative Themes

##### High quality engagement

Participants discussed experiencing positive affect as well as instrumental benefits during strengths profiling, for example, “I like how we can all just have a bit of a laugh, but at the same time have a good think about how I’ve been with my feelings and that and trying to get them under control.” There were also reports of participants losing track of time and engaging in rich and passionate conversations about their character strengths. One facilitator explained, “there were a lot of good insights from the young people. I also noticed that they were all asking lots of questions throughout the session and they genuinely seemed really engaged with the activity.” This engagement was aided by finding a balance between the written work and group discussion, for example, “I thought it would just be like, sitting in a room all day and just filling out paper work and that, but it’s not… So that’s good” (young person).

##### Autonomy supportive

The degree of autonomy experienced by participants was another key factor in supporting their engagement in strengths profiling. Young people attended voluntarily, with various motivations for being there, from simply “passing the time because there was nothing else to do” to developing confidence for a job interview (e.g., “I just wanna be more confident in myself and to be able to not be scared to go for like interviews”). The facilitators did not dictate the goals of strengths profiling and instead encouraged young people to set their own goals and direct the activity toward meaningful areas of their lives. For example, one facilitator talks of their experience with a group of young mothers, “When brainstorming the strengths, the young people came up with much more suggestions when we started thinking about what strengths are needed to look after their babies and young children.” Facilitators also gave “options for the strengths profiles [e.g., the number of strengths listed, how to graphically display the discrepancy scores etc.] to give them choice in how they want to engage in the activity” (facilitator). The discussions around character strengths also came about organically, with another facilitator explaining how “often a discussion broke out about mental skills or sometimes on subjects completely unrelated, which we encouraged.” Young people were given the space to gauge their own involvement, where “some worked more quietly than others, which I don’t think was them not being engaged, just them taking a lot of time to think about it” (facilitator).

##### Strengths-based self-reflection

Young people welcomed the opportunity to reflect on, set goals, and develop their vocabulary around their character strengths. Participants regularly voiced how the structured reflection surrounding their character strengths that was led by the facilitators was not something they typically engaged in prior to the program, for example, “You evaluate the skills you use, the skills you’re good at and the skills you’re not good at. And then you look at how you can work on these. So I think it’s good because it’s not something you necessarily always think about.” Several young people also voiced their satisfaction with the strengths-based approach to reflection, and how it helped them to look toward a brighter future, for example, “I’ve been knocked down and stuff and drawing a line under it and moving on and getting into things that I need to get myself into. So, I’m quite happy, it keeps me in my mind happy.” A facilitator later noted that a support worker had commented “that for some of them [young people] it was the first time that he had heard them talking about their aspirations.” In addition, young people left the session feeling inspired to incorporate new ways of reflecting, for example, “2 days later now, when I’m on my own, I’ll have a good think about what I’ve done today. It does make me feel a bit more happier in myself.”

##### Relevance and accuracy

A theme discussed by almost every participant was how important and accurate they felt their strengths profiles were, usually surpassing their prior expectations. Young people felt their top character strengths (i.e., those with the lowest discrepancy scores) were indeed ones they used on a daily basis and were key to current and future success. For example, “getting my point across. I think that’s pretty good for different parts of life because like, you gotta get your point across, no matter what it is, in everyday conversation, in debates, with life, staff here, or anything.” Young people also felt those who knew them best would agree with their profiles; for example, “being courageous, I mean, ask anybody which knows me well enough. [I] basically get a little bit nitty gritty, do the things that other people might shy away from.”

##### Individuality in meaning

Often, it was only when hearing a young person describe their character strength that it became clear what that characteristic was referring to. Young people would often use broad terms in reference to very specific characteristics; for example, one young person included problem-solving as one of their top strengths, which to them referred specifically to their financial situation, “going to the shop working out your money or your income, your rents, your bus fares here there and everywhere, and like what you’re gonna be doing working.” At times, a characteristic meant very different things to different young people; for example, one young person defined confidence as being comfortable in “what you’re wearing,” whereas another used confidence in reference to speaking “to a lot of different people every day.”

##### Social influences

Participants discussed a number of ways that being in a group supported their process of strengths identification. Most commented on having enjoyed the interactions with their peers, and for others, attending the group felt challenging but was seen as a way of improving their social anxiety (e.g., “[I attended] to try and get out there more, because at the moment I do have anxiety, so me being in a group like this has pushed quite a limit, but that’s why I keep coming because it’s building on that”). Young people also reported it useful to “learn a little bit more about other peoples’ mental strengths and skills and how they cope with things” (young person), which supported them in identifying their own character strengths. A shared understanding between young people often meant they “chipped in with noting each other’s strengths, which was nice” (facilitator). At times, being aware of each other’s strengths also helped to build connections, particularly when a young person expressed a quality that others felt in need of; for example, “being caring. I apply that to my life by just being there for people, even if they’re not there for me, you know, I’m always door open, heart-warming.” Often, the young people attributed their comfort with the social environment and ease in which they were able to discuss their character strengths to the positive rapport they had built with the facilitators, who were described as able to “get through to the [young people] which not many people can do” (young person).

##### Protective factors

The majority of young people did not have the support systems typical of those their age, and compounding this issue was the complex needs and mental health difficulties many were grappling with on a daily basis. It was apparent that, for most young people, it was their perceptions of signature character strengths that served as their main, and sometimes only, source of security. For example, one young person talked about how optimism was their signature strength, “If you don’t have optimism, if you don’t have hope for a better day each day, you’re just gonna feel depressed.” Other young people described how having perspective enabled them to keep going, “I use this when I’m recovering from daily stresses and I will tell myself that I’ve made it through worse.” or how their positive attitude enabled them to “bounce back from things, even when I’ve been through some difficult things I’m always smiling, trying to make the better situation out of what it is.”

#### Quantitative Data

##### Discrepancy score

The quantitative data was in line with the qualitative themes in that young people scored their characteristics highly in importance and ideal level, which remained high post-course (Table 3). In support of the aforementioned theme of protective factors, negative correlations were also observed between discrepancy scores and measures of resilience, self-worth, and well-being, ranging from small to moderate in effect size, both at baseline and post-intervention (Table 4).

**TABLE 3:** Pre-to post-program changes in discrepancy scores, resilience, self-worth, and well-being.

**Variable**	***n***	**Mean pre (range)**	**SD pre**	**Mean post (range)**	**SD post**	***t* or *F***	***P***	**95% CI**	**Effect size**
**Strengths profile**									
Importance	48	9.23(3.11-10)	0.87	9.32(4.08-10)	1.01	*t* = 0.50	0.616	−0.27 to 0.44	*d* = 0.10
Ideal level	48	9.23(6.60-10)	0.86	9.37(7.33-10)	0.66	*t* = 1.11	0.271	−0.11 to 0.39	*d* = 0.12
Current level	48	6.59(1.55- 8.75)	1.55	7.87(3.18- 9.71)	1.33	*t* = 7.25	< 0.001^∗^	0.93 to 1.65	*d* = 0.89
Discrepancy	48	23.43(2.46- 83.74)	15.78	14.32(0- 50.44)	12.10	*t* = *−*5.57	< 0.001^∗^	−12.40 to −5.82	*d* = 0.65
**Resilience**	50	3.34(1.70- 4.90)	0.73	3.67(2.20- 5)	0.74	*t* = 3.43	0.001^∗^	0.13 to 0.51	*d* = 0.45
**Self-worth**	47	3.27(1.54- 4.92)	0.82	3.47(2.23- 4.81)	0.78	*t* = 2.47	0.017	0.03 to 0.30	*d* = 0.25
**Well-being**									
Engagement	51	2.98(1- 5)	0.92	3.24(1.75- 5)	0.86	*F* = 8.79	0.005^∗^	0.03 to.42	η^2^ = 0.17
Perseverance	51	3.27(1.92- 5)	0.77	3.57(1.25- 5)	0.84	*F* = 3.53	0.067	−0.03 to.53	η^2^ = 0.07
Optimism	51	3.06(1- 5)	1.02	3.35(1.75- 4.75)	0.84	*F* = 3.75	0.058	−0.01 to.47	η^2^ = 0.07
Connectedness	51	3.54(1.75- 5)	1.01	3.76(1- 5)	0.94	*F* = 3.78	0.058	−0.06 to.43	η^2^ = 0.08
Happiness	51	3.12(1.5- 5)	0.85	3.25(1.57- 5)	0.94	*F* = 1.69	0.200	−0.08 to.34	η^2^ = 0.04

*A Bonferroni corrected alpha value of 0.008; ^∗^*p* < 0.008; SD, standard deviation; CI, confidence interval.*

**TABLE 4:** Correlations between discrepancy scores and measures of resilience, self-worth, and well-being.

	**1**	**2**	**3**	**4**	**5**	**6**	**7**	**8**
**(a) Baseline for the full sample (*n* = 118).**								
(1) Discrepancy score	1							
(2) Resilience	−0.31^∗∗^	1						
(3) Self-worth	−0.44^∗∗∗^	0.61^∗∗∗^	1					
(4) Well-being (Engagement)	−0.25^∗∗^	0.46^∗∗∗^	0.35^∗∗∗^	1				
(5) Well-being (Perseverance)	−0.31^∗∗^	0.61^∗∗∗^	0.47^∗∗∗^	0.42^∗∗∗^	1			
(6) Well-being (Optimism)	−0.38^∗∗∗^	0.66^∗∗∗^	0.69^∗∗∗^	0.56^∗∗∗^	0.60^∗∗∗^	1		
(7) Well-being (Connectedness)	−0.22^∗^	0.39^∗∗∗^	0.35^∗∗∗^	0.46^∗∗∗^	0.39^∗∗∗^	0.46^∗∗∗^	1	
(8) Well-being (Happiness)	−0.35^∗∗∗^	0.63^∗∗∗^	0.57^∗∗∗^	0.63^∗∗∗^	0.54^∗∗∗^	0.75^∗∗∗^	0.59^∗∗∗^	1
**(b) Baseline for the subsample who completed a post-intervention measure (*n* = 49).**								
(1) Discrepancy score	1							
(2) Resilience	−0.39^∗∗^	1						
(3) Self-worth	−0.49^∗∗^	0.56^∗∗∗^	1					
(4) Well-being (engagement)	−0.24	0.61^∗∗∗^	0.35^∗^	1				
(5) Well-being (perseverance)	−0.25	0.56^∗∗∗^	0.45^∗∗^	0.35^∗^	1			
(6) Well-being (optimism)	−0.34^∗^	0.63^∗∗∗^	0.68^∗∗∗^	0.55^∗∗∗^	0.41^∗∗^	1		
(7) Well-being (connectedness)	−0.23	0.54^∗∗∗^	0.38^∗^	0.58^∗∗∗^	0.41^∗∗^	0.54^∗∗∗^	1	
(8) Well-being (happiness)	−0.39^∗∗^	0.66^∗∗∗^	0.52^∗∗∗^	0.70^∗∗∗^	0.41^∗∗^	0.74^∗∗∗^	0.66^∗∗∗^	1
**(c) Post-intervention subsample (*n* = 49).**								
(1) Discrepancy score	1							
(2) Resilience	−0.28	1						
(3) Self-worth	−0.48^∗∗^	0.62^∗∗∗^	1					
(4) Well-being (engagement)	−0.30^∗^	0.47^∗∗∗^	0.42^∗∗^	1				
(5) Well-being (perseverance)	−0.20	0.49^∗∗∗^	0.23	0.56^∗∗∗^	1			
(6) Well-being (optimism)	−0.21	0.57^∗∗∗^	0.56^∗∗∗^	0.54^∗∗∗^	0.56^∗∗∗^	1		
(7) Well-being (connectedness)	−0.12	0.36^∗^	0.29^∗^	0.47^∗∗^	0.61^∗∗∗^	0.53^∗∗∗^	1	
(8) Well-being (happiness)	−0.36^∗^	0.69^∗∗∗^	0.57^∗∗∗^	0.75^∗∗∗^	0.55^∗∗∗^	0.70^∗∗∗^	0.60^∗∗∗^	1

*Discrepancy score = (ideal − current) × importance; ^∗^*p* < 0.05, ^∗∗^*p* < 0.01, and ^∗∗∗^*p* < 0.001. No correlations were observed between discrepancy score and any of the demographic variables measured.*

From pre- to post-program, the only significant and meaningful changes in the strengths profile scores were those for current level and discrepancy score (Table 3). This change in discrepancy score from pre-to post-program was examined in the reduced sample size (*n* = 41 to 47) and resulted in no significant correlations with pre- to post-program changes in resilience, self-worth, and well-being, although the correlation with the change in self-worth was of a small effect size (*r* = 0.27, *p* = 0.09). In addition, a significant, positive correlation was found between the change in discrepancy score and the duration of time the participant had been staying in the sheltered accommodation (*r* = 0.32, *p* = 0.03).

##### Classification of strengths

Each participant listed an average of 10.27 characteristics (*SD* = 3.23) within their strengths profile. Based on the meanings provided by participants, 217 different character strengths were identified across the sample, each with multiple terms used to describe it. The vast majority (98%) of the 217 character strengths identified were deductively categorized into the 24 VIA subcategories. Only four terms (“water,” “images,” “thorority [*sic*],” and “preferable person”) could not be categorized due to ambiguous wording and inadequate description obtained to explain what the terms referred too. This process revealed an average of 7.03 VIA character strengths per profile (*SD* = 1.82), meaning that participants often included more than one term within their strengths profile to describe the same VIA group of character strengths (e.g., “focused,” “hardworking,” and “committed” all featured in the same profile and referred to “perseverance” according to the participant’s definitions).

**FIGURE 1:**
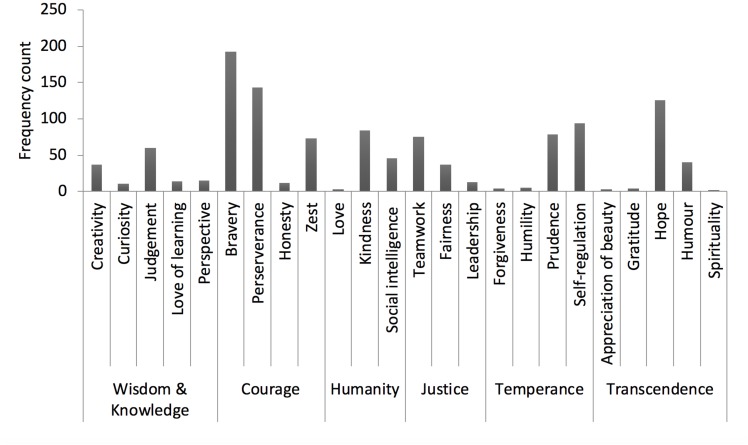
The frequency each of the 24 VIA character strength groups featured in the strengths profiles.

Figure 1 shows the frequency each of the 24 VIA character strength groups featured within the strengths profiles, with strengths related to bravery, perseverance, and hope listed most often. Bravery alone comprised 22 variations, with “confidence,” “mentally tough,” and “courage” being the most frequently included. Perseverance also comprised 22 variations, with “maintaining focus,” “ability to adapt,” and “commitment” being the most frequent. Hope comprised 19 variations, with having a “positive attitude,” “optimism,” and “belief” being the most frequent (see online Supplementary Material for the full list of variations in character strength labels).

## Discussion

This study aimed to investigate the experiences of homeless young people when using strengths profiling to identify character strengths. Participants were found to engage well with strengths profiling as suggested in both the qualitative and quantitative data. Every young person who attended the activity willingly completed a strengths profile, despite it being framed as optional. In addition, participants provided rich information surrounding their character strengths, with an average of over seven VIA characteristics identified. In a comparable study, Tweed et al. (2012) used conventional interviews to explore what homeless adults felt were their character strengths and identified an average of just over one VIA character strength per participant. These findings suggest that the lack of character strengths in homeless young people identified in previous research could, in part, be explained by the participants’ engagement with the assessment approach or the terminology used to define character strengths (Heinze, 2013).

Participants of the present study also felt that their strengths profiles and discrepancy scores were accurate and meaningful reflections of their character strengths. Although the facilitators encouraged participants to consider whether some characteristics felt more important than others, and to score their ideal level realistically in terms of a score out of 10 that felt achievable and satisfactory given their current level, the young people typically scored all their characteristics extremely high in both importance and ideal level (i.e., ≥0.9). This finding may be expected given that the characteristics were closely related to their day-to-day sense of safety and were self-selected from a large pool. In contrast to importance and ideal level, scores for current level showed greater variance between characteristics, participants, and time points, and therefore had a much stronger correlation with discrepancy scores. This may raise the question as to whether the scores for importance and ideal level were therefore redundant. Statistically, this argument is somewhat supported in the data; however, at an individual, qualitative level, scores for importance and ideal level provided valuable information for young people and the facilitators, such as to get to know each other better, build rapport, and tailor the intervention. For example, individuals often had multiple characteristics with the same current score, in which case, even slight differences in scores for importance and/or ideal level (e.g., 9/10 vs. 10/10) resulted in different discrepancy scores and facilitated goal setting. Further to this, in other populations, where strengths profiling may be directed toward other areas of life (e.g., job performance), the variance in scores for importance and ideal level may be greater. It was for these reasons that a score for importance was first added to performance profiling used in sports settings (Jones, 1993).

In line with prior research, a more positive perception of character strengths (i.e., lower discrepancy scores), was negatively related to feelings of resilience, self-worth, and well-being (e.g., Seligman et al., 2005; Hutchinson et al., 2010; Proctor et al., 2011). In addition to highlighting the informative nature of strengths profiling, this finding also reinforces the benefit of using a strengths-based approach with homeless young people who typically report lower well-being, self-worth, and resilience compared to the general population (Heinze, 2013).

In support of the VIA framework (Peterson and Seligman, 2004), the vast majority (98%) of character strengths identified through strengths profiling could be deductively mapped onto one of the 24 groups of VIA character strengths. A high level of agreement was anticipated given that strengths profiling was directed to psychological strengths of character (aka “mental strengths/skills”), which are what underpins the extensive and well-established VIA framework (Peterson and Seligman, 2004). Indeed, previous studies that have reported identifying “non-VIA” strengths are typically referring to strengths related to physical and occupational skills (e.g., construction and “being good with hands;” Tweed et al., 2012), rather than strengths of character. In addition, the 24 groups of character strengths within the VIA framework were found to be broad enough to accommodate a wide range of terms and individual meanings.

As predicted by Kelly’s personal construct theory (PCT; Kelly, 1955), participants in the present study were unique in their labeling and description of character strengths. The process of transformation therefore required careful consulting of the participants’ individual meanings behind a label used to decipher which VIA category it best suited. Often a label appeared to fit a VIA category but, when considering the participant’s meaning, it instead suited a different one. At other times, although a participant’s definition suited to a particular VIA character strength in the eyes of the researcher (*post hoc*), it is unlikely this connection would have been made by the participant. For example, one participant used the label “organic” to describe themselves as being authentic and having integrity, yet, although their description was matched very closely to the VIA character strength “honesty,” the young person rejected this alternative term during discussions. The term organic, in this case, had a unique meaning for the participant.

In addition to these unique labels and meanings, and in line with the individuality corollary of PCT (Kelly, 1955), the present findings also show that participants only had a select few character strengths they considered relevant to their current situation. That is, participants included an average of seven VIA character strengths within their strengths profiles on which to rate themselves, only 29% of the possible 24. Conceptually, one might argue that it is a more engaging, autonomous, and positive experience to score yourself on a selection of characteristics you have identified as being meaningful to you, rather than in areas imposed by a third party (Deci and Ryan, 1985). For example, an individual who does not value spirituality, may lose interest and motivation when forced to rate themselves in this area, only to then be informed that they have not scored highly. Autonomy is indeed a possible mechanism for the high engagement observed in the present study, which is also supported by previous studies in athletes that have found performance profiling to be associated with high levels of intrinsic motivation (Weston et al., 2011). However, it is important to note that in focusing on the character strengths considered important by the participant, strengths profiling is at risk of missing important characteristics that the individual may not be immediately aware of, such as strengths that have the potential to become important in the future, or strengths that are valued by significant others in their social system. These considerations are why the VIA-IS (Peterson and Seligman, 2004) has an important place in character strength research, and strengths profiling should therefore be considered as an alternative approach that maps well onto the VIA conceptually. If strengths profiling is used in isolation, the skills of the facilitator are particularly important during the initial brainstorming phase of strengths profiling, where the group discussion is guided to as many potential areas of character strength as possible. As reflected in the qualitative data, this brainstorming process was effective in developing character strength awareness and vocabularies, before opportunities were provided during MST4Life^TM^ to use and further develop these strengths. In future practice, it may be useful to incorporate the VIA framework into this guided reflection phase of strengths profiling to maximize on the characteristics considered.

With regards to the frequency each of the 24 VIA character strength groups featured in participants’ strengths profiles (Figure 1), the findings map very closely on to the prevalence of VIA character strengths identified in a related but slightly older sample of homeless adults living in supported accommodation (Tweed et al., 2012). Similarly, Tweed et al. (2012) found perseverance, kindness, and social intelligence to be some of the most endorsed strengths, whereas curiosity, humility, appreciation of beauty/excellence, forgiveness, and gratitude were less frequently mentioned. Interestingly, although gratitude was not frequently reported within strengths profiles, young people regularly expressed their gratitude for the intervention, both in diary room entries and to the facilitators. The biggest difference between Tweed et al. (2012) and the present study is that the younger sample within the present study placed much greater importance in bravery, hope, and teamwork. Previous research has shown a relationship between a person’s character strengths and their age. For example, character strengths such as appreciation of beauty and excellence, forgiveness, and judgment are less common among young children and adolescents (e.g., Park and Peterson, 2006a, b; Park et al., 2006), a finding that was also evident in the present study (Figure 1). These findings again support the idea that individuals may benefit from self-selecting the character strengths that are most relevant to their stage of life.

The present study also revealed strengths profiling to be sensitive to detecting change and, similar to performance profiling in sport (Weston et al., 2013), may therefore be used to monitor fluctuations in character strengths over time, such as when evaluating interventions. Although the aim of this study was to explore the feasibility of strengths profiling, rather than to evaluate the youth development program it was used within (MST4Life^TM^), the study nevertheless provides support for this broader intervention. Significant and meaningful improvements were observed in character strength discrepancy scores and perceptions of resilience, self-worth, and well-being. Previous interventions have similarly demonstrated an ability to improve perceptions of character strengths in other populations (e.g., Catalano et al., 2004; Quinlan et al., 2012); however, character strengths are considered relatively trait-like and stable across time and are only reactive to highly meaningful experiences (Park and Peterson, 2009). It is therefore encouraging that perceptions of character strengths can be bolstered in homeless young people over the course of a 10-week intervention. However, given the lack of control group, attribution of these outcomes in the present study remains tentative pending further evidence. Nevertheless, qualitative results revealed that the character strengths identified during strengths profiling were crucial protective factors in the lives of these homeless young people, which explains why the development of these areas through MST4Life^TM^ is likely to have positively impacted resilience, self-worth, and well-being. This finding is particularly significant during a time when levels of homelessness are critically high, with the situation further compounded by decreased funding streams, and affordable strengths-based interventions recommended as a preventative and reactive solution (Farrow et al., 1992; Centrepoint, 2015; Homeless Link, 2016).

### Future Research and Applied Practice

Future research in positive psychology and character strengths may wish to further consider the integration of ideographic and nomothetic instruments and the effect each has on strengths-based assessment and intervention. As previously mentioned, protective factors in homeless young people were embedded in their perceptions of their character strengths and the unique and contextualized meanings attached to them. The ideographic strengths profiling tool enabled these young people to take ownership and work with their idiosyncratic and highly contextualized character strengths over the course of the intervention. In contrast, nomothetic surveys transform these ideographic qualities into generic character strength labels and definitions. Some argue that this nomothetic and prescriptive approach to identifying character strengths can alienate individuals from their true experiences, similar to that of the medicalized approach used in the diagnostic and statistical manual of mental disorders (DSM) (McDonald and O’Callaghan, 2008). Previous research that has made direct comparisons between ideographic and nomothetic measures in related fields has focused solely on the measurement accuracy and degree of overlap (or lack of) (e.g., Schiller et al., 1995; Robazza et al., 2000; Van Kampen, 2000; Grice, 2004). An area that has received little attention is a comparison of participant experiences during the process of strength identification, such as perceptions of autonomy and intrinsic motivation to use and further develop the strengths identified. Differences in such experiences may impact the resulting awareness of signature strengths and subsequent strengths use and well-being.

Whilst ideographic approaches may prove more beneficial in understanding and developing personal constructs during character strength interventions, nomothetic approaches are often necessary in achieving statistically generalizable research (Holt, 1962; Runyan, 1983). Indeed, to identify statistical generalizations and address nomothetic hypotheses, it is helpful to have a measure such as the VIA-IS that is standardized and easily comparable across individuals and groups (Dunn, 1994; Grice, 2004). Another option is to use a mixed-method approach, where nomothetic and ideographic methods are employed together (e.g., questionnaires and interviews), which allows the researcher to generalize from group aggregates, whilst at the same time particularize from individual cases (Runyan, 1983; Morse, 2016). However, a limitation of mixed-methods is the extensive data collection, processing, and analyses required, which is not always possible in applied practice, where researchers, practitioners, and frontline staff typically prefer single tools that are practical and easy to implement and interpret (e.g., Mackeith, 2010). To address this issue, the present study demonstrates that ideographic and nomothetic lines of enquiry can be addressed within a single tool. The data from strengths profiles, which was inherently entwined in an individual’s personal constructs, could be unfolded *post hoc* and with a high degree of accuracy, into the nomothetic VIA framework. In doing so, this broadens the scope of strengths profiling by enabling standardized comparisons across individuals and groups, thus addressing the statistical generalizability limitations of ideographic approaches (Runyan, 1983). Strengths profiling could, therefore, be recommended and further explored as an intervention and research tool for the collection of both ideographic and nomothetic data. It is important to note, however, that in the present study, the transformation from ideographic strengths profiles into the VIA framework was carried by experienced researchers. As a result, this transformation may not always have been understood or agreed upon by the participants. This type of transformation should therefore be reserved for *post hoc* research purposes should nomothetic questions be required, rather than as a way of trying to change an individual’s personal constructs during an intervention into the language of the VIA, as such an approach would go against the aforementioned benefits of an ideographic and person centered approach.

Another area for consideration in future research is the social environment in which character strengths are identified. In the present study, and in line with the sociality corollary of PCT (Kelly, 1955), young people found the social environment to play a vital part in the success of strengths profiling. Aligned with previous research into the identification of character strengths in young people (e.g., Steen et al., 2003), participants in the present study helped each other to identify and label their character strengths, which was aided by a shared understanding of their unique life situations. These social interactions are particularly important in disadvantaged young people, where positive youth development interventions typically aim to build social connections and interpersonal skills (Scales et al., 2000). With many character strength interventions now being conducted online and/or with individuals in isolation (Quinlan et al., 2012), further research is needed to explore the impact of individual vs. group environments on character strengths identification and use.

## Conclusion

Overall, the present study demonstrates the utility of strengths profiling in a population of homeless young people. This person-centered approach enabled voices to be heard that may not have come forward in other more conventional research approaches (Curtis et al., 2004). These homeless young people, who have rarely featured in previous character strengths research, engaged well with strengths profiling and provided rich accounts of their individual character strengths. The resulting strengths profiles comprised unique and highly contextualized character strength labels and definitions, which served as vital and meaningful protective factors in their lives. Identifying character strengths in this way enabled those strengths to be discussed and developed over the course of the intervention in a way that preserved each young person’s unique meaning and attachment to their signature strengths. The later categorization of strength profiling data into the VIA framework also demonstrated that the benefits of the VIA conceptualization can be obtained through strengths profiling. The effectiveness of strengths profiling has significance for various researchers and practitioners whom require a pragmatic and easy-to-use approach to strengths-based assessment and intervention. After having provided initial support for the utility of strengths profiling, further research in now required to directly compare this approach with other nomothetic tools and explore the effect each has on the experience of strengths identification and subsequent use.

## Ethics Statement

This study was carried out in accordance with the recommendations of the British Psychology Society Code of Human Research Ethics with written informed consent from all subjects. All subjects gave written informed consent in accordance with the Declaration of Helsinki. The protocol was approved by the University of Birmingham Ethics Committee.

## Author Contributions

SC and JC conceived the study idea and designed the research. SC, MQ, and BJ collected and processed the data. SC analyzed the quantitative and qualitative data, and wrote the first draft of the manuscript. JC, MH, MQ, and BJ contributed to the interpretation of the data. All authors contributed to the manuscript revision, and read and approved the submitted version.

## Conflict of Interest Statement

The authors declare that the research was conducted in the absence of any commercial or financial relationships that could be construed as a potential conflict of interest.

## Footnotes

1 https://stbasils.org.uk

## Appendix A

### Strengths Profile Worksheet

**Table:**

**Characteristic**	**Meaning**	**Importance**	**Ideal Level**	**Current Level**	**Discrepancy Score**
1.					
2.					
3.					
4.					
5.					
6.					
7.					
8.					
9.					
10.					
11.					
12.					
					

*Strengths profiling was adapted from performance profiling used in sport (e.g., Butler and Hardy, 1992; Jones, 1993); Discrepancy score = (Ideal level − Current level) × Importance.*
