# Supplementary material for: The Experiences of Homeless Youth When Using Strengths Profiling to Identify Their Character Strengths
Source: Front Psychol. 2019 Sep 24;10:2036. doi: 10.3389/fpsyg.2019.02036 (PMC6769124; doi:10.3389/fpsyg.2019.02036)
Supplement: Supplementary file 1 [file Table_1.DOCX]

**List of synonyms and variations attributed to each of the VIA character strength groups^[[1]](#footnote-1)^**

| **VIA character strength** | **Number of variations** | **Variations** |
| --- | --- | --- |
| Creativity | 2 | creativity, intuitive |
| Curiosity | 6 | challenge yourself, curiosity, inquisitive, open-minded, openness, receptive |
| Judgement | 16 | analytical skills, changing thoughts, common sense, concentration, initiative, logic, managing expectations, perceptive, prioritizing, problem solving, realistic, realistic expectations, realistic goals, reflection, tactical, thoughtful |
| Love of learning | 10 | active, clever, intelligent, keen to learn, love of learning, math, obtaining GCSE’s, productive, quick learner, self-efficient |
| Perspective | 5 | awareness, awareness of surroundings, explanation, insightful, perspective |
| Bravery | 22 | ability to change, ability to cope, aggression, assertiveness, bold, bravery, coping with change, confidence, courage, direct, fearless, independent, mentally tough, positive self-talk, pride, resilience, self-belief, self-pride, self-regard, speaking in groups, standing up for myself, strong |
| Perseverance | 22 | adaptability, attentiveness, commitment, concentration, consistent, determination, disciplined, drive, effort, endurance, flexibility, focused, goal orientated, independence, motivated, overcoming problems, perseverance, persistence, resolve, willpower, work hard, working under pressure |
| Honesty | 5 | being myself, honestly, integrity, organic, outspoken |
| Zest | 11 | anger/passion, attitude, can do attitude, competitiveness, determination, energetic, enthusiastic, chilled, motivated, on it, to go the extra mile |
| Love | 4 | closeness, good wife, good parent, loving |
| Kindness | 16 | attitude, being nice, calming, caring, compassionate, empathetic, friendly, helpful, kindness, listener, outgoing, patient, sharing, supporting others, trustworthy, understanding |
| Social intelligence | 8 | chatty, communication, engaging, eye contact, interactive, persuasive, social skills, talkative |
| Teamwork | 12 | assertive, communication, contribution, cooperation, loyalty, punctual, professional, reliability, responsible, take one for the team, team building, teamwork |
| Fairness | 5 | fairness, respect for others, manners, self-orientation, self-respect |
| Leadership | 3 | leadership, management, team leader |
| Forgiveness | 2 | forgiveness, regretful |
| Humility | 4 | down to earth, grounded, humble, self-awareness |
| Prudence | 18 | avoid laziness, balance, being realistic, decision making, distraction control, control diet, control sleep, good choices, help yourself, keep fit, know limits, money management, mindful, organization, pacifist, time management, treat self, work-life balance |
| Self-regulation | 13 | anxiety control, control, control energy levels, coordination, cope with stress, emotional control, manage temper, motivation, positive self-talk, relaxing, self-regulation, stay calm, stress free |
| Appreciation of beauty | 3 | amazing, appreciation, love life |
| Gratitude | 3 | grateful for life, grateful for others, recognize achievements |
| Hope | 19 | ambition, belief, defined goals, dreams, futuristic, gaining independence, goal setting, good mindset, high expectations, hope, imagery, motivation, optimism, planning long-term, positive attitude, positive image, seeing goals ahead, think forward, vision |
| Humor | 5 | cheerful, enjoyment, fun, happy, humor |
| Spirituality | 3 | being a mother, evolution, make a change |

1. As discussed in the article, the terms presented were attributed to their respective VIA character strength based on the individualized definition provided by each participant and not by the term itself. Some terms therefore featured across multiple VIA character strengths. Participants’ definitions were often very different to the term used and can be provided on request to the authors. [↑](#footnote-ref-1)
